# Extensive estuarine sedimentary storage of plastics from city to sea: Narragansett Bay, Rhode Island, USA

**DOI:** 10.1038/s41598-023-36228-8

**Published:** 2023-06-23

**Authors:** Victoria M. Fulfer, J. P. Walsh

**Affiliations:** 1 Graduate School of Oceanography, University of Rhode Island, Narragansett, RI 02882 USA; 2 Coastal Resources Center, Graduate School of Oceanography, University of Rhode Island, Narragansett, RI 02882 USA

**Keywords:** Ocean sciences, Environmental sciences

## Abstract

Plastics are an important new component of the global sedimentary system, and much concern exists about their transport, fate and impact. This study presents the first system-scale assessment of sedimentary storage of microplastic for an estuary, Narragansett Bay, RI (USA), and the measurements of shoreline and seabed sediments add to the growing body of literature demonstrating high coastal concentrations. Microplastic concentrations in sediments ranged from 396 to over 13,000 MP particles kg^−1^ dry sediment (DW), comparable to other shoreline and seafloor sites located near urban centers. As previously reported for fine sediment and other pollutants, estuarine plastic storage is extensive in Narragansett Bay, especially within the upper urbanized reaches. Over 16 trillion pieces of plastic weighing near 1000 tonnes is calculated to be stored in surface sediments of the Bay based on a power-law fit. This work highlights that estuaries may serve as a significant filter for plastic pollution, and this trapping may have negative consequences for these valuable, productive ecosystems but offer potential for efficient removal.

## Introduction

Plastics are an important new component of the global sedimentary system, observed on mountain tops and in ocean trenches, and are among the most ubiquitous forms of pollution in coastal and marine environments around the world^1–3^. Sedimentary plastics are a tracer for the Anthropocene in the geological record and raise concerns about negative impacts on organisms and ecosystems^4–7^. The fate of plastics is anticipated to differ from siliciclastic and carbonate sediments due to their lesser and variable density, and the distribution and movement of marine plastics is a thriving body of research^8–10^. Microplastics are particles smaller than 5 mm in size^11,12^, and thus cover a range from gravel to mud. They can enter the environment as primary microplastics, e.g., pellets used in plastic production and cosmetic products^11^, or as secondary microplastics from fragmentation of larger plastics^8^. Estimates of plastic material flux to the ocean vary from 0.41 to 4 million metric tons annually^12–14^. However, only 1% of the marine plastic is estimated to remain in the water column^10,15^. Most is believed (70–99%) to be deposited on shorelines and in marine sediments^15,16^. The objective of this study was to examine sedimentary plastics along a city-to-sea gradient of a large estuarine system, Narragansett Bay (NB), Rhode Island, USA to inform deposition and storage on shorelines and the seabed.

### Background and study area

Estuaries are home to valuable ecosystems, providing critical habitats that are important spawning habitat for marine biota, storm surge protection and also areas for ports, marine businesses and more^17^. Due to their circulation, wetlands and other processes, estuarine areas serve as significant traps for sediments^18,19^, carbon^20–22^ and pollutants^23^. Unlike deltaic systems, most fine sediment transported into estuaries will accumulate and only a small fraction may move seaward^24^. Microplastics are hypothesized to follow a similar pattern, based on past studies demonstrating high estuarine MP concentrations^25^. Sources of microplastic pollution to estuaries can include runoff, fisheries, wastewater treatment facilities, shipping, industrial plastic production, and littering of single-use plastic items^26^. Many microplastics entering estuarine environments will be deposited along with sediments on shorelines or the seafloor, due to their excess density relative to seawater (e.g., PVC) or increased density due to biofouling, aggregation, or incorporation into fecal pellets^27,28^.

**Figure 1:**
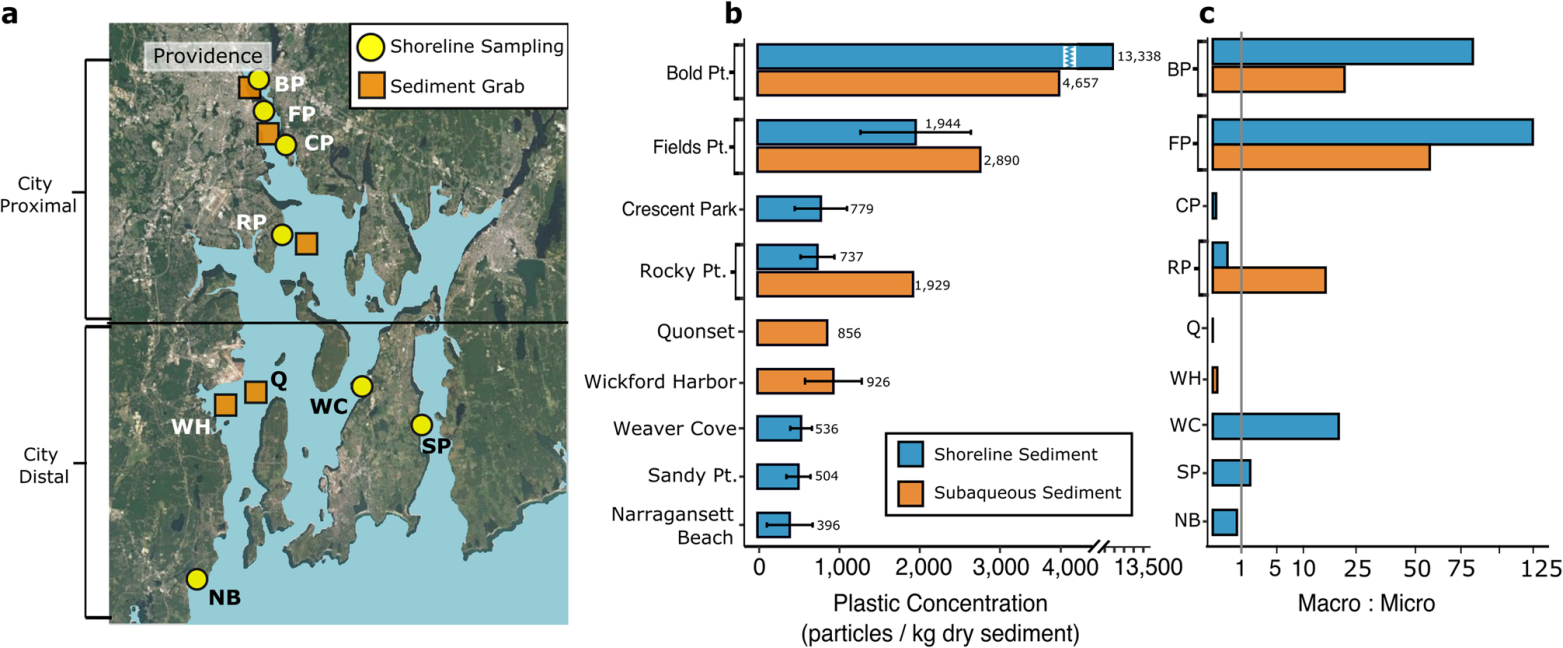
Locations of shoreline (yellow circles) and subaqueous sediment grab (orange squares) sampling sites in Narragansett Bay (**a**). Each shoreline site included 2–3 transects within which the upper beach, mid-beach, intertidal zone, and subaqueous zone were sampled. At each sampling location, 94–100% of all plastic measured were microplastics (< 5 mm). Plastic concentrations (particles/kg dry sediment) range from about 400 to over 13,000 particles/kg dry sediment (**b**). Macroplastics dominate by mass (g plastic/kg dry sediment) and the ratio of macroplastic to microplastic shows that macroplastic mass is greater in the upper bay and decreases near the lower estuary (**c**). Satellite image derived from Google Earth v7.3.6.9345 (December 14, 2015; https://earth.google.com/)) and edited using a grid from Global Multi-Resolution Topography Synthesis (GMRT)^70^.

NB is a large (342 km^2^), partially- to well-mixed estuary with a north–south orientation (Fig. 1a)^29,30^. Average water depth is ~ 9 m, and in general, it has a counterclockwise surface circulation^31,32^. This system is subject to wind, wave, tidal, runoff and human forcings^33^. During nor-easters and hurricanes, gale force winds, storm surge and associated waves (sea and swell) affect aeolian and hydrodynamic transport, and semi-diurnal tides (< 1.5 m) influence the currents^34^. Mean flushing time of the Bay is 26 days^34^, and human modifications to the system are widespread.

NB is carved out of the Narragansett Basin sedimentary strata and its morphology and stratigraphy have been heavily influenced by glacial processes^35,36^. Fine sediment input to the system is very low due to the low-sediment yield rivers draining to the estuary, and many fluvial sources are dammed upstream^35^. Sediment accumulation in NB is controlled by microtidal estuarine sedimentation processes which deposit fine sediment in deep, sheltered areas in the upper and middle Bay, and sandy sediment dominates the mouth of the Bay due to ocean transport by waves and near-bed currents^36^. Modern day net sediment accumulation rates in NB are low, generally < 3 mm year^−1^^23,37–39^.

The NB coast has a dense urban population, particularly in the “Proximal” zone near Providence, situated at the head of the estuary (Fig. 1). Development in the watershed and along shoreline was significant during the industrial revolution^40^, and as a result, NB is well-known for its pollution history, with previous work highlighting heavy metals and organic pollutants^23,30,39,41,42^. Today, plastic pollution is an increasing concern as it is apparent during any visit to the Bay, and like other estuaries, it poses an unknown threat to the ecosystem including micro- and macro-organisms and humans that consume seafood from the system^5,43–46^. The specific aim of this study was to determine the distribution and concentrations of microplastic particles in shoreline and seafloor sediment of NB with the hypothesis that plastic concentrations will decrease down the estuary and major storage exists.

### Estuarine storage is dramatic

**Figure 2:**
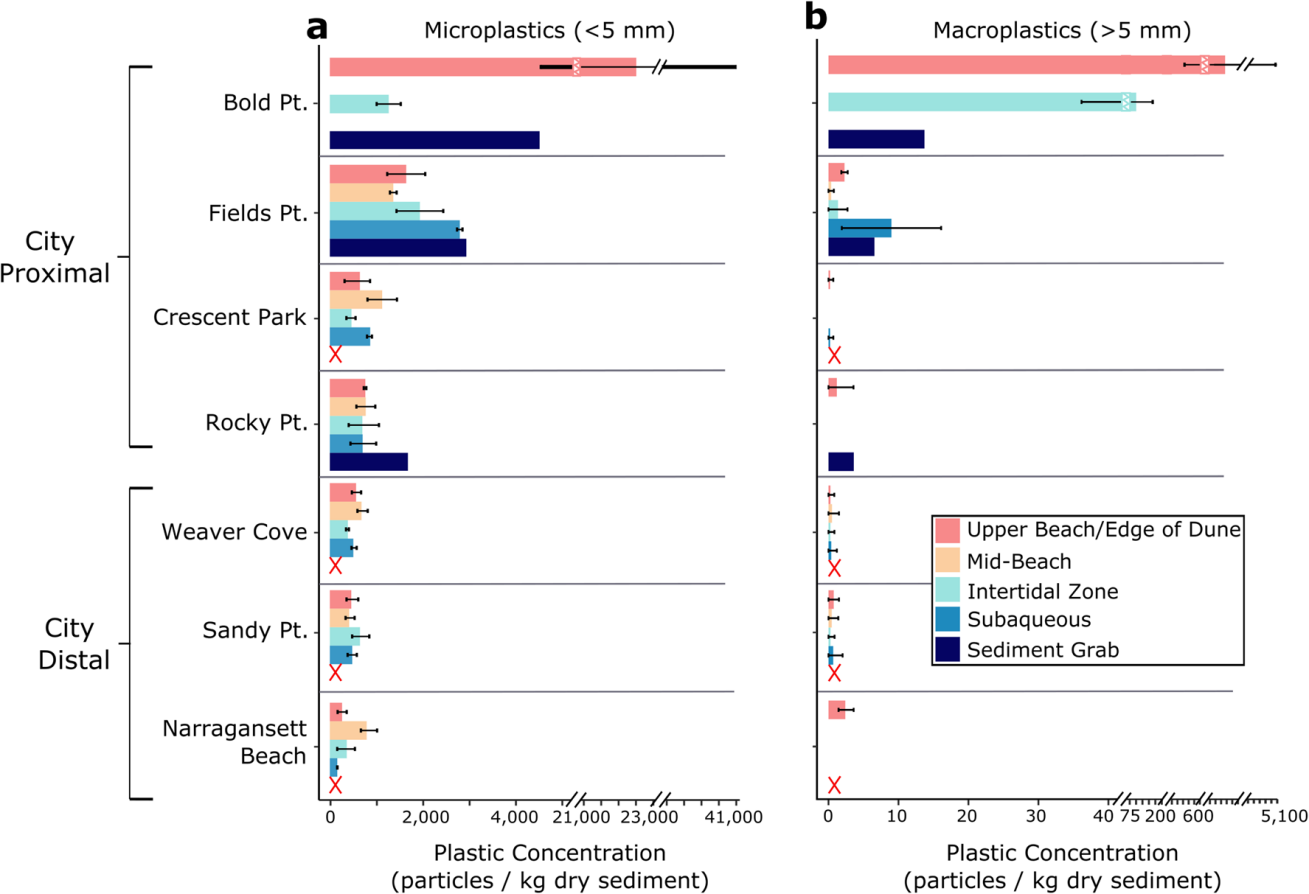
Plastic concentration (particles/kg dry sediment) is high in the upper estuary and decreases toward the open ocean for both microplastics (**a**) and macroplastics (**b**). Variability is observed within sites when comparing samples taken in the upper beach (pink), mid-beach (orange), intertidal zone (aqua), and subaqueous zone (blue). Sediment grab samples located offshore of shoreline sites are included for comparison (navy). Red ’X’ denotes where no samples were taken. Error bars represent one standard deviation from the mean.

**Figure 3:**
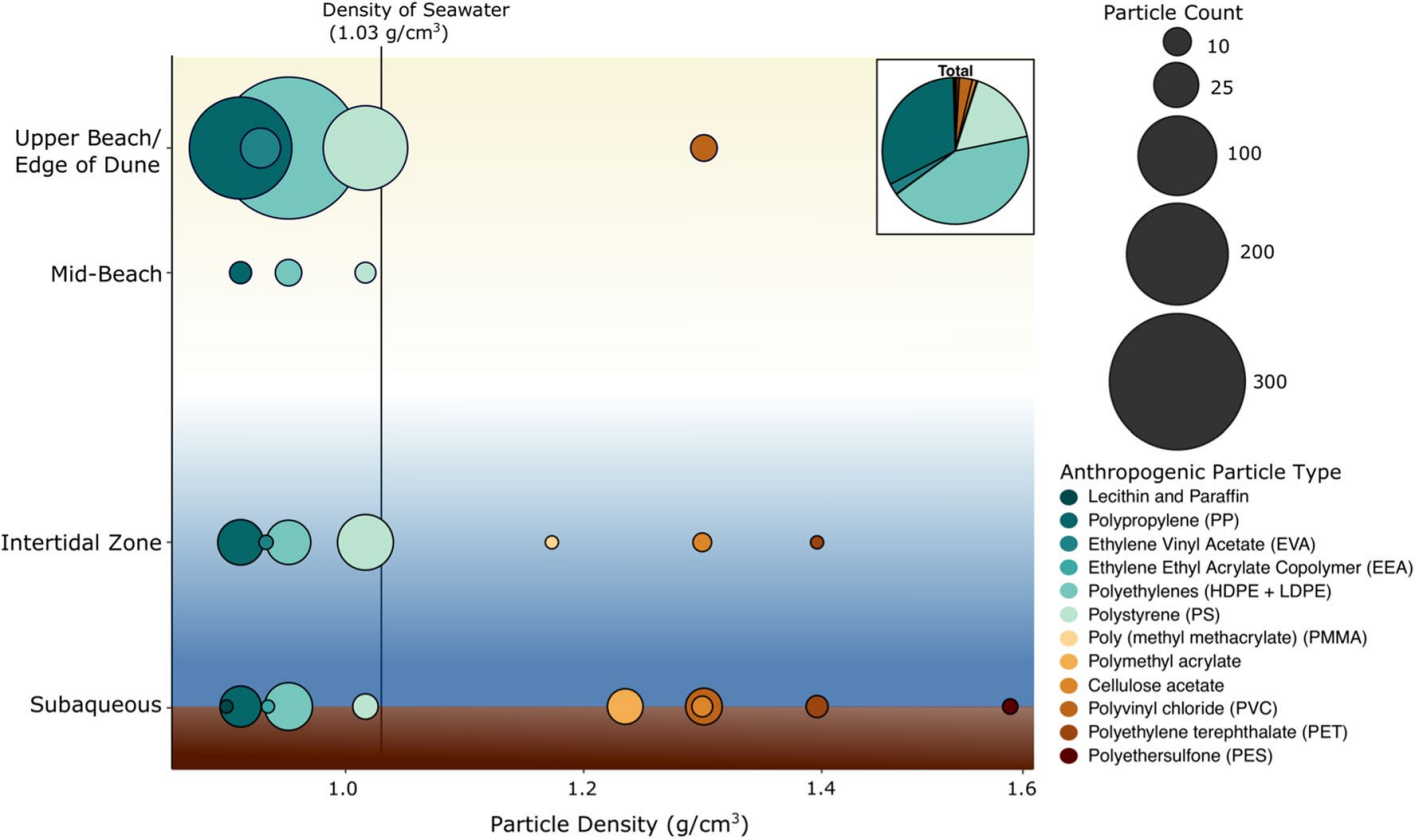
Abundance of microplastic particle types varied between sampling zones (e.g., upper beach, mid-beach, intertidal zone, subaqueous). The size of the dot represents the microplastic particle count. The color represents both the particle type and the corresponding particle density. Particles less dense than seawater (< 1.03 g cm^−3^) are shown in green and blue shades. Particles more dense than seawater (> 1.03 g cm^−3^) are shown in shades of brown. For polymers with a range of possible densities (e.g., polystyrene), the average density was used. The overall frequency for all sites and sampling zones is shown in the “Total” pie chart in the top righthand corner.

Microplastics have been found to be nearly ubiquitous in both coastal and open ocean marine environments, from polar seas to the equator^8–11,47–51^, and not surprisingly, microplastics were found in all NB sediment samples, at varying concentrations (Figs. 1 and 2), with measurements and particle types consistent with published data on subaqueous microplastic concentrations^52^. The measurement methodology detailed below was robust, involving care against contamination, polymer determination and testing of extraction efficiency and error, but differences in sampling protocols and research methodologies (e.g., MP particles kg^−1^ DW; particles L^−1^ sediment; g km^−2^; particles m^−2^) limit direct comparisons with other studies. Shoreline and seafloor sediment from the Proximal Zone had high mean MP concentrations, ranging from 1944 to 13,338 MP particles kg^−1^ DW (Fig. 1b). A down-estuary decreasing trend was evident, but cross-shore patterns were not as other work has suggested^53,54^. Along- and across-shore variability is expected due to differences in coastal zone current circulation cells, beach morphology and wave run-up^55,56^ (Fig. 2). However, aside from the very high shoreline plastic concentrations found at Bold Point, NB seafloor sediments (mean 856–2890 MP particles kg^−1^ DW) had consistently higher values than those from the shoreline (mean 396–1944 MP particles kg^−1^ DW), and this is despite the fact that most particles consisted of polymers that are less dense than water (Fig. 3), suggesting particle biofouling and aggregation are important and rapid enough to allow significant proximal trapping. The average concentration of MPs in shoreline sediment in the Distal Zone ranged from 396 to 536 MP particles kg^−1^ DW. These values are comparable to other sandy beaches in the United States. Sediment samples from estuarine and barrier islands of Virginia and North Carolina contained 600–2200 MP particles kg^−1^^57^, and southeastern USA beach MP concentrations ranged from 40 to 450 MP particles kg^−1^ DW^58^.

**Figure 4:**
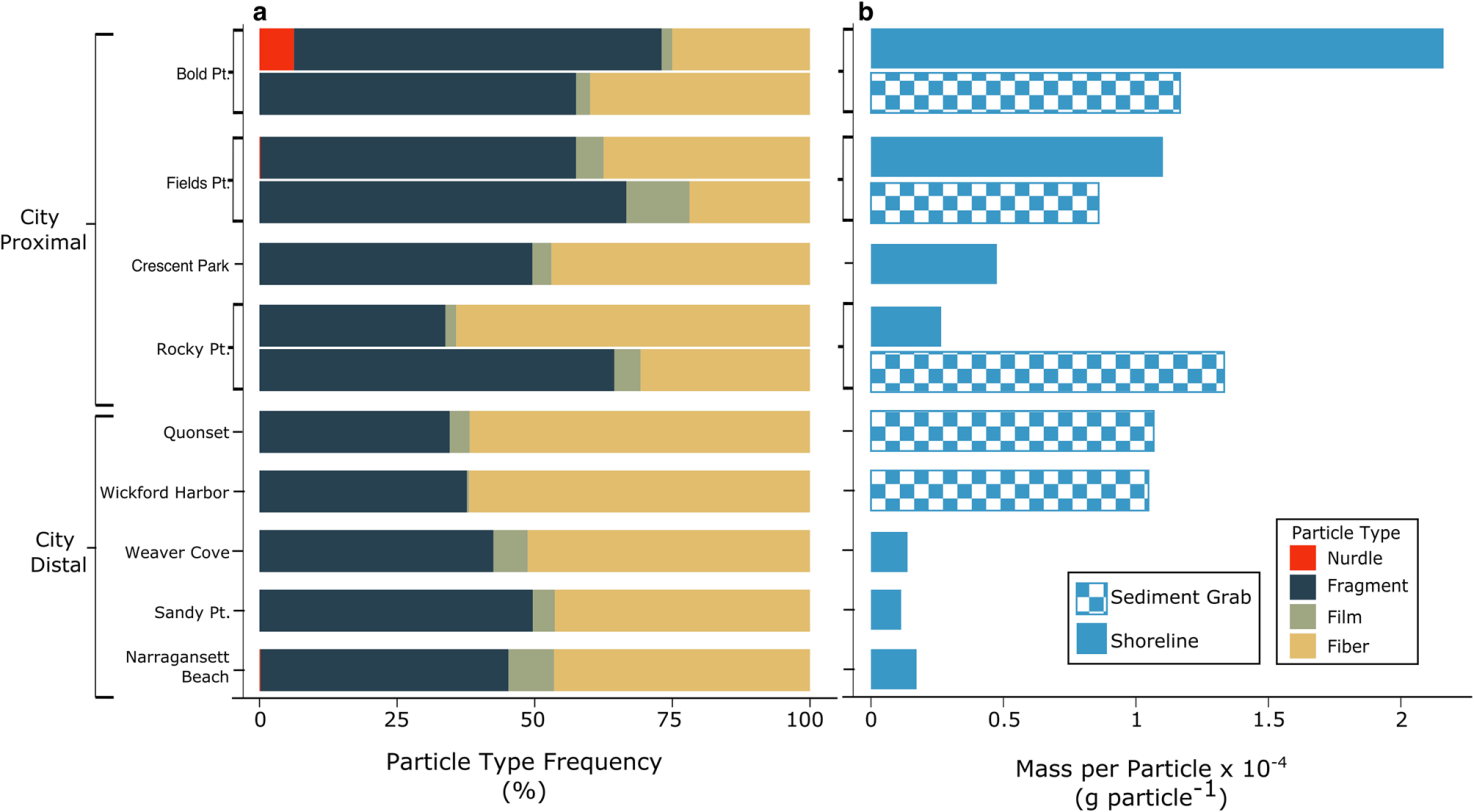
Plastic particle types include pre-production pellets or nurdles (red), fragments (navy), films (green), and fibers (yellow). Fibers and fragments dominate by frequency (%; **a**). Mass per particle (g particle^−1^) decrease down the system in shoreline samples (solid bars) but remain consistently high in sediment grab samples (checked bars; **b**).

Microplastics in NB sediments were found to be abundant and diverse in size, shape, and polymer type (Figs. 3 and 4). While all samples contained microplastic particles, only 68% contained macroplastics (> 5 mm). Interestingly, the occurrence of microplastics did not positively correlate with macroplastics. The Proximal Zone contained more macroplastic than microplastic by mass (macro : micro > 1), while the opposite was true for most sites in the Distal Zone (macro : micro < 1; Fig. 1c). This inverse observation could indicate that macroplastics are being beached or settling out of the water column in the Providence River and upper NB, thus not reaching the sites in lower NB. Additionally, these macroplastics are likely being degraded and broken down into microplastics during the transport process from the Proximal to the Distal zone, and those microplastics are more easily transported seaward^59,60^.

Many studies have shown that fibers are the dominant form of microplastic in coastal environments in the USA and around the world^4,6,49,58,61,62^. In this study, fragments and fibers dominated at each site, with fiber abundance accounting for 21–70% of all microplastics identified (Fig. 4). Low and high density polyethylene (LDPE, HDPE; 43%), polypropylene (PP; 32%) and polystyrene (PS; 17%) were the most common plastic types found in NB sediments (Fig. 3). This is unsurprising, as 90% of worldwide plastic production is for LDPE, HDPE, PP, polyvinyl chloride (PVC), and PS^58^. Particle shape and polymer type likely play important roles in transport and storage of microplastics in the NB system, and more investigation is needed in this regard.

### City-to-sea sedimentary storage of microplastics

**Figure 5:**
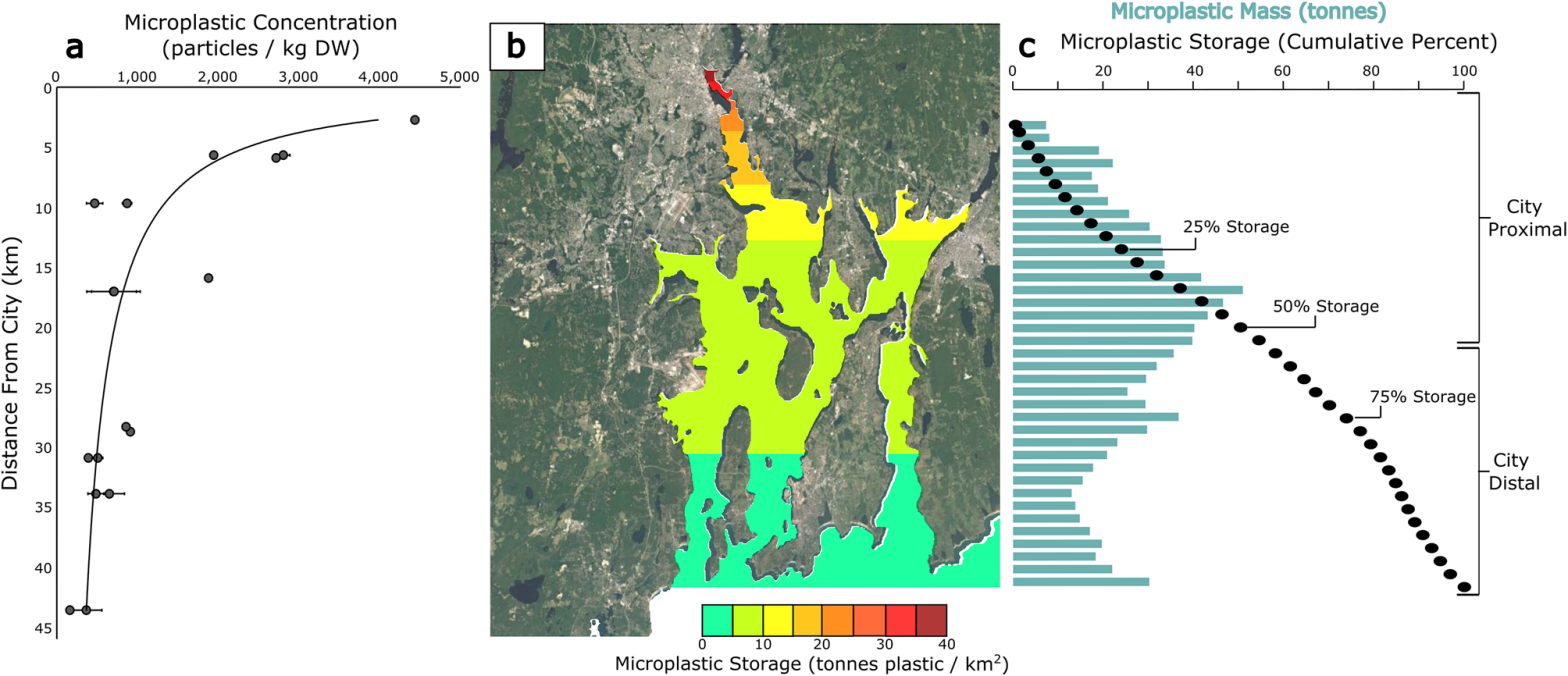
A negative power-law relationship between microplastic concentration (particles kg^−1^ dry sediment) in subaqueous sediment and the distance from Providence, RI (km) exists (R^2^ = 0.85; **a**). Error bars represent one standard deviation from the mean. Some error bars are sufficiently small to be contained within the circle symbol. Using this relationship, the depth of the sediment sampled (top 5 cm), the average density of the sediment (1.7 g cm^−3^) and the average mass per plastic particle (5.86 × 10^−5^ g particle^−1^), the predicted amount of plastic contained in any given area of the Bay is calculated. This is mapped onto a grid of Narragansett Bay (**b**). When the total area of the bay is summed, the mass of microplastics contained in the top 5 cm of subaqueous sediment totals 9.76 × 10^5^ kg, or 976 tonnes. Over 50% of microplastics are estimated to be stored in the upper Bay proximal to the city (**c**). Satellite image derived from Google Earth v7.3.6.9345 (December 14, 2015; https://earth.google.com/) and edited using a grid from Global Multi-Resolution Topography Synthesis (GMRT)^70^.

A dramatic, down-system decreasing gradient of plastic concentrations was observed in both shoreline and seafloor sediments of NB (Fig. 1b). Highest values are from the head of the Bay (Bold Point Park in the Providence River), and lower but still significant concentrations were measured at the most seaward site, Narragansett Town Beach, near the mouth. The decreasing trend was also apparent in MP concentration by mass (g plastic/kg dry sediment) and in the mass ratio of macro- to microplastic (Fig. 1c). Pollution research has noted that a power-law relation to urbanization is common^63,64^, and data here fit well to this type of model (R^2^ = 0.85; Fig. 5a). This trend is expected due to the high population and urbanization, and thus increased possible sources along the Proximal Zone. Population densities are over 1930 people per km^2^, in the Proximal Zone and are an order of magnitude lower in the Distal Zone^65^. Several studies have shown high MP concentrations in coastal areas adjacent to cities^66,67^. For three Australian estuarine systems, Hitchcock and Mitrovic^68^ found microplastic abundances generally relate to the human activity, and a study of Chesapeake Bay showed a weak (r = 0.33) positive linear correlation between surface water MP abundance and population density^69^. Current predictions of global plastic input from rivers to the ocean rely on geographically limited water-column concentration data and lack the data needed to account for plastic deposition in estuaries^12,14^. To better understand the plastic problem (impact and storage), power-law relationships may be used as a tool to model plastics with other proxies (e.g., population or land-use patterns) to estimate coastal sources and fluxes^63^.

A wealth of studies have shown that estuaries act as filters for material entering the system from rivers, including sediments, organic matter, nutrients, and pollutants^21,22,71^. However, this is the first study to demonstrate substantial, system-wide storage as hypothesized by other studies^62,72–76^, and this is likely the case in other estuaries. The effectiveness of estuarine sediment trapping has been related to many spatially and temporally varying processes including flocculation as well as estuarine circulation and wetland storage. The high amount of microplastic deposition in the Proximal Zone (> 50%) indicates that a large fraction of plastic entering the Bay is, at least initially, stored in subaerial and subaqueous deposits near the population center.

Based on the power-law fit (Fig. 5), nearly 1 × 10^3^ tonnes of microplastics are estimated to be trapped in the top 5 cm of NB sediment, representing over 16 trillion particles assuming an average weight of 5.8 × 10^−5^ g/particle. This rough estimate provides a first-order look at the magnitude of the plastic pollution problem in a single urbanized estuary. The plastic flux into the Bay from a multitude of sources is very difficult to quantify. Work by Lebreton et al.^12^ estimated coastal MP flux based on concentrations in river waters, and calculated plastic input to the ocean from all rivers to be 1.15–2.41 million tonnes per year^12^. However, these estimates do not take into account the substantial estuarine sediment trapping as estimated here, and thus new approaches to constrain plastic land-to-sea transfer are needed. A modeling study of Chesapeake Bay calculated that 94% of riverine microplastics are deposited in the sediment, with the remaining 5% exported from the estuary and 1% remaining in the water column^77^. Based on the river plastic input predictions by Lebreton et al.^12^ and the estimation of 94% deposition in sediments^77^, up to 14 tonnes of plastic per year could be deposited in NB sediment from river input alone, likely an underestimate given other additional sources of MP to NB (coastal littering, WWTF, fishing, etc.). Given this, much if not most of the plastic input to the ocean may be stored, at least temporarily, in estuarine sediments. With this in mind, in addition to reducing inputs around the world, there is an opportunity to attack the plastic problem by more regularly cleaning the estuarine plastic filter; some efforts have focused on the water column, but more work is needed along the estuarine shorelines and seabed. It is worth noting that this study also measured very high plastic concentrations in dredged material from the distal end of the fluvial network at the head of the Bay. Concentrations ranged from 5600 to 11,900 MP particles per kg DW sediment. These data further support the power-law relationship observed, and highlight that lower river storage is also critical. Accordingly, dredged material management should be carefully monitored to limit the reintroduction of plastics.

### Sedimentary environments and microplastics

MP concentrations are reportedly highest in low-energy, depositional sedimentary settings, such as lagoons and fjords^25^; These locations also trap fine-sediment and are often zones of accumulation for metals and organic pollutants. In this study, sediment grain size varied dramatically between sites, reflecting local conditions as noted by McMaster et al.^78^, and most samples consisted of a mix of fine sand to gravel (granules) (Supplementary Fig. S2). Sites located in the Proximal Zone had the highest percentages of mud (7–44%) compared to all other sites (0–2.5% mud) and contained the highest concentrations of MPs. Other work has shown positive correlation between microplastics and finer sediments^50,79,80^. Also, sampling transects at Field’s Point, a protected depositional basin at the head of the estuary, exhibited a notable fining trend from the upper beach to subtidal sediments, and this fining trend coincided with a strong increase in MP concentration (Supplementary Fig. S3; Fig. 2). These data help illustrate how estuarine geomorphic pockets can enhance sediment and plastic storage, but such features also make accurate budgets challenging to achieve.

With high concentrations in coastal sediments, there is much concern about how plastics may impact organisms and ecosystem functioning. Everaert et al.^81^ combined literature data on potential effects of microplastics on infauna and has proposed 540 particles kg^−1^ sediment as a potential threshold for harm. In NB, about 80% of the measurements currently exceed this value. Because rivers with large sediment loads may dilute MP concentrations, it is possible that NB sediment has more elevated concentrations than other systems. In contrast, sediment accumulation rates in Manila Bay, Philippines vary from 1 to 9 cm year^−1^^82–84^. Because seven of the top ten rivers in the world that contribute plastic pollution are located in the Philippines^14^, it could be expected that MPs in this area would be extremely high. However, one study near five river mouths of Manila Bay found concentrations of only 386–1357 particles kg^−1^ dry sediment^85^. Even at the upper end, these concentrations are 30–70% lower than the MPs from upper NB. This difference may be explained by dilution as well as differing measurement methods or transport efficiencies. The influence of sediment supply relative to plastic input should be further studied.

Particle density, surface area and size also play a role in the deposition of microplastics, and thus will impact the correlation between sediment grain size and MP concentrations^86,87^. Of all MPs analyzed in this study, 95% had densities less than seawater (< 1.03 g cm^−3^). Particles more dense than seawater were found almost exclusively in the intertidal and subaqueous samples. Other studies have similar findings, with on average 92.2–95.8% of measured plastics being low density plastics like PE, PP, and PS^88–92^. Biofouling must be important in this and other estuaries as it can increase sinking rates by an order of magnitude per day^28,93,94^. In NB, the down-estuary increase in fiber abundance and decrease in fragment abundance supports the idea of fragment fouling and storage resulting in down-system decrease in mass per plastic particle (g particle^−1^) seen in the shoreline sites (Fig. 4). This study did not examine biofouling on individual particles, but other work has shown rapid biofouling rates. Bacteria in coastal sediments colonized LDPE particles after just 14 days, and PE films rapidly developed biofilms in seawater after 3 weeks^95,96^. Given this, biofouling is believed to be critical to the observed pattern of MP in NB.

In sum, this first system-wide study of estuarine sediments demonstrates extensive trapping of MP, providing additional evidence that coasts bear additional burden of high microplastics. These observations indicate significant and far-reaching ecosystem consequences, but they also offer an opportunity for more efficient plastic removal to help limit broader impact on the ocean.

## Methods

### Sampling

#### Shoreline transects

Shoreline transects were carried out at seven sites between September and October of 2020, with one site (Bold Point, RI) sampled in March 2021 (Supplementary Table S1). Sites were chosen to provide a spatial distribution ranging from the Lower Providence River to the mouth of Narragansett Bay. At each site, three transects perpendicular to the shoreline were completed within 2 h of low tide. Along each transect, four sample locations were chosen: (1) the upper beach, near the dune toe; (2) mid-beach; (3) intertidal zone, between the high tide line and edge of the swash zone; and (4) a subaqueous sample taken approximately 50 m from the shoreline (Supplementary Fig. S1). The wrack line was not sampled at any site.

Separate sediment samples were collected for grain-size analysis, macroplastic measurement and microplastic analysis. Samples were limited to the upper 5 cm of sediment. All sediment was collected in pre-rinsed glass jars using a metal spoon. Cotton clothing was worn during collection.

#### Seafloor sediment samples

To evaluate seabed plastics, sediment grab samples were collected at five locations throughout Narragansett Bay (Supplementary Table S1, Supplementary Fig. S1). Samples were collected using a Van Veen grab sampler deployed from the *R/V Cap’n Bert* on two occasions, in Fall 2020 and Summer 2021. Separate sediment samples were collected for grain-size analysis, macroplastic analysis, microplastic analysis and an archive sample. Sediment was collected in pre-rinsed glass jars using a metal spoon.

### Plastic extraction and analysis

Shoreline and seabed sediment samples were processed identically. One liter of sediment was wet sieved at 1 mm using metal sieves. Debris > 1 mm were dried in an oven at 60 °C for 24 h. Debris was then transferred to a large glass pan and any suspected macroplastics (> 5 mm) or large microplastics (1–5 mm) were removed using metal forceps. Plastics were transferred to a glass petri dish for further analysis. A description of each particle along with the mass, color, and plastic type (e.g., film, fiber, fragment, foam, pellet) was recorded.

The plastic research literature analyzes plastics at a wide range of sizes, and there is a methodological limit to the size of particles that can be confidently measured (approximately < 40 microns). Given the sedimentological focus of this study, this study decided to set a lower limit of 62.5 microns, the sand-mud size cutoff for sediments, for plastics to analyzed as this is a standard sieve size used for geological analyses and can confidently be measured. This lower size limit may limit broader comparisons to other studies, which apply a range of size cutoffs including at 38 µm^50^, 45 µm^52^, or 100 µm^4^.Approximately 100 g of wet sediment was weighed and sieved at 62.5 µm to remove fine sediment. The remaining sediment was transferred to a 500 mL glass beaker for microplastic extraction. The sieve was then inspected under the dissecting microscope to ensure no fibers or other particles had been left behind. A dense solution of sodium iodide (NaI, 1.8 g/cm^3^) was used for extractions, following procedures from Kedzierski et al.^97^.

The NaI solution was added until the volume of NaI was twice the volume of sediment. The beaker was covered with aluminum foil and stirred for 5 min using a glass stir bar. The solution was left to settle for a minimum of 6 h and a maximum of 24 h. Once the NaI solution was clear, the supernatant containing any floating particles was carefully decanted through a 62.5 µm sieve, avoiding resuspending the sediment. The sieved NaI was poured back into the sediment sample, stirred for 5 min, and left to settle for a second extraction. The particles caught in the sieve were rinsed into a small glass beaker using pre-filtered (0.2 µm) DI water.

Organic digestions were carried out on Bold Point Park samples, which were from a marshy shoreline and thus contained a high presence of organic material in the post-extraction material. An aliquot of 2 mL of 30% H_2_O_2_ was added to the beaker containing the extracted material and allowed to digest overnight at 60 °C. All extracted material was filtered through a pre-weighed 47 mm diameter, 1.6 µm pore size GF/F filter using vacuum filtration with a glass filter funnel. The filter was then placed in a small glass jar for future analysis. Once dry, filters containing extracted material were weighed.

Filters were examined using light microscopy under a dissecting microscope, with all suspected plastic particles counted and categorized into fragments, fibers, and films. Fluorescence was also used to help locate plastic particles on the filter. NIGHTSEA royal blue (440–460 nm excitation) illuminated the filter, which was viewed using the dissecting microscope fit with a 500 nm longpass emission filter (NIGHTSEA SFA Stereo Microscope Fluorescence Adapter). When possible, plastic particles were removed from the filter using fine forceps and adhered to a glass slide using double-sided tape. Plastic particles were imaged at 40X zoom using an AmScope dissecting microscope fit with an Amscope MU1803 digital camera. The size of particles > 500 µm was measured using ImageJ (Rasband 2018)^98^.

#### Extraction efficiency

To verify efficacy of the extraction methods, fragments of polystyrene and polyethylene terephthalate, low density polyethylene film, as well as polypropylene fibers, were created using a coffee grinder followed by sieving to attain a representative particle size distribution and imaged and sized using ImageJ (Supplementary Table S1). Sediment samples were spiked with a known number of particles and extracted as described above. Extraction efficiency following the second extraction ranged from 77.1 to 100%, with a mean efficiency of 92%.

#### Polymer Identification

Fourier-transform infrared spectroscopy (FTIR) was used to determine the polymer makeup of all particles able to be picked by forceps, typically > 250 µm in size (Shimadzu IRTracer-100). Sample spectra were collected over the range of 500–4000 cm^−1^, with a data interval of 1 cm^−1^ and resolution of 4 cm^−1^. The ATR diamond crystal was cleaned with 70% 2-propanol and a background scan was performed between each sample. All collected spectra were compared to the Shimadzu spectra libraries for identification. A minimum match of 80% was required for spectra to be accepted as plastic particles.

### Sediment analysis

Approximately 50 g of wet sediment from each sample location were weighed and dried in an oven at 60 °C for 24 h, and reweighed. The moisture content of each sample was calculated using the wet and dry weights. Grain size of each sediment sample was measured using a Malvern Mastersizer 3000. Samples were first sieved at 2 mm to remove the gravel fraction, which was corrected for in the final distribution determination.

### Contamination controls

100% cotton clothing was worn during sampling, and a cotton lab coat was worn during all laboratory procedures. All extractions were performed under a laminar flow hood, which was cleaned before each extraction began. Plastic materials were avoided at all possible steps of sampling and extraction, and all glassware was prerinsed. Filters were placed in the laminar flow hood to serve as air blanks during each extraction. Filters were also collected every 12 samples to test the DI water and NaI solution for contamination. All particles found on blanks were recorded (Supplementary Table S2). If a particle of the same color and shape was found in the blank and sample, corrective action was taken and that particle was subtracted from the total count for that sample. The maximum number of particles on any single blank was 4, and 49% of counted filters had at least one particle removed through blank subtraction (Supplementary Table S3).

### Estimating plastic mass in Narragansett Bay sediments

Using microplastic abundances from subaqueous and intertidal zone shoreline samples, as well as subaqueous sediment grab samples, a negative power-law relationship between microplastic abundance (particles kg^−1^ dry sediment) in subaqueous sediment and the distance from the city of Providence, RI (km) was found (R^2^ = 0.85)(Fig. 5a). Using this relationship and Eq. (1), we predict the amount of plastic contained in any given area of Narragansett Bay.

1 $$P_{mass\_total} = \mathop \sum \limits_{2.7}^{42} \left[ {\left( {9450.4\; \times \;d^{ - 0.861} } \right)\; \times \;\left( {Az{\uprho }_{s} } \right)} \right]\; \times \;m$$

In Eq. (1), *z* represents the depth of the sediment sampled (top 5 cm), *m* is the average mass per plastic particle (5.86 × 10^−5^ g particle^−1^), *d* is the distance from Providence (km), *A* is the seafloor area of each grid cell, and ρ_s_ is the density of sediment (1.7 g cm^−3^). By summing the plastic contained in the subseafloor area from 2.7 km (the distance of our first sample site) to 42 km (the distance to the mouth of the Bay), we predict the total amount of plastic contained in the top 5 cm of subaqueous sediment to be 9.76 × 10^5^ kg, or 976.3 tonnes, of microplastic. Microplastics are not distributed evenly in Narragansett Bay sediment (Fig. 5C). 25% of the total plastic mass, or approximately 240 tonnes, are contained in the upper 12% of the Bay area, or the area north of Rocky Pt., Warwick, RI. The area north of Quonset, RI represents 60% of the entire Bay area, but holds 75% of the microplastics.

## Supplementary Information

Supplementary Information.

## Data Availability

The data presented in this manuscript have been submitted to the Zenodo open science data repository (https://doi.org/10.5281/zenodo.7696956).
