# Supplementary material for: Extensive estuarine sedimentary storage of plastics from city to sea: Narragansett Bay, Rhode Island, USA
Source: Sci Rep. 2023 Jun 23;13:10195. doi: 10.1038/s41598-023-36228-8 (PMC10290116; doi:10.1038/s41598-023-36228-8)
Supplement: Supplementary file 1 — Supplementary Information. [file 41598_2023_36228_MOESM1_ESM.pdf]

Extensive Estuarine Sedimentary Storage of Plastics from City to Sea: Narragansett Bay, Rhode Island, USA

Victoria M. Fulfer[1,2*] and J. P. Walsh[1,2]

*Corresponding author: Victoria M. Fulfer; vmf713@uri.edu
[1]Graduate School of Oceanography, University of Rhode Island, Narragansett, RI 02882
[2]Coastal Resources Center, Graduate School of Oceanography, University of Rhode Island, Narragansett, RI 02882

Supplementary Figures and Tables

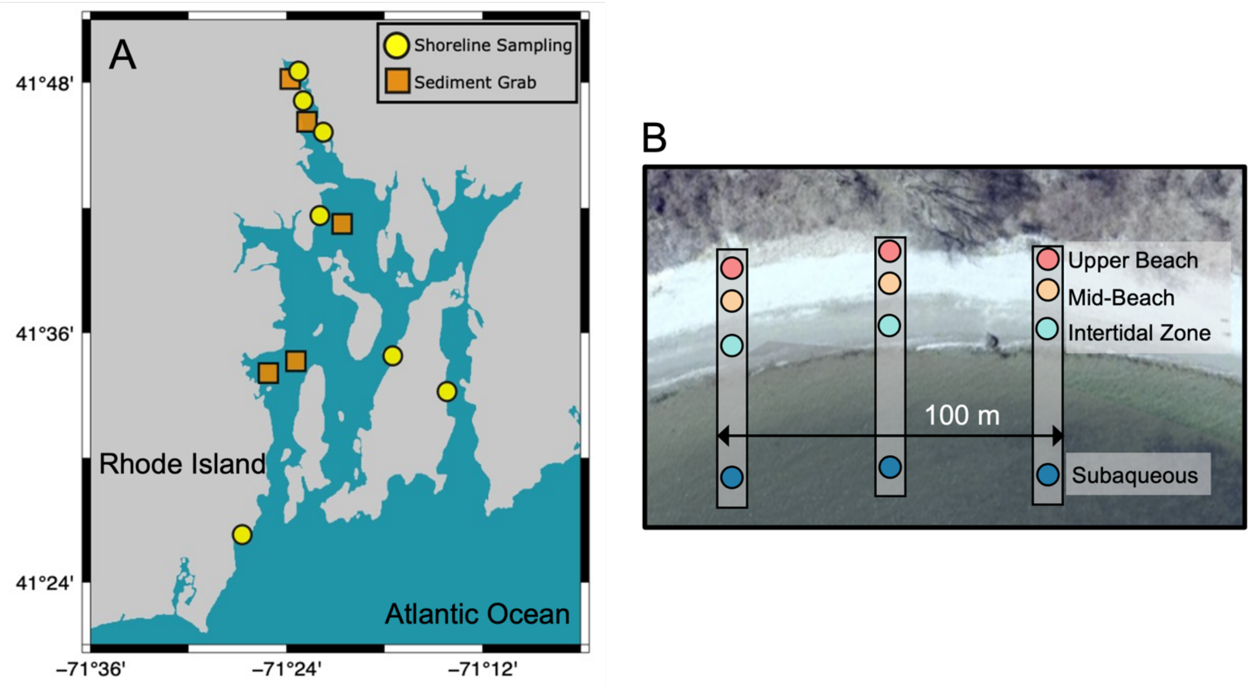

Supplementary Figure S1. Locations of shoreline (yellow circles) and subaqueous sediment grab (orange squares) sampling sites in Narragansett Bay (A). Each shoreline site included 2-3 transects (B) within which the upper beach, mid-beach, intertidal zone, and subaqueous zone were sampled. Map was created using a grid from Global Multi-Resolution Topography Synthesis (GMRT)[100].

Supplementary Table S1. Study Site Locations

Site	Abbreviation	Date	Sample Type	Latitude	Longitude
Bold Pt. Park, Providence	BP	3/22/21	shoreline survey	41° 48' 30.7794"	-71° 23' 22.074"
Bold Pt. Park, Providence	BP	6/3/21	sediment grab	41° 48' 26.64"	-71° 23' 42.54"
Field's Point, Providence	FP	10/1/20	shoreline survey	41° 47' 13.7148"	-71° 22' 48.0504"
Field's Point, Providence	FP	6/3/21	sediment grab	41° 43' 44.76"	-71° 21' 18.8994"
Crescent Park, Riverside	CP	10/8/20	shoreline survey	41° 45' 29.016"	-71° 21' 51.1194"
Rocky Point, Warwick	RP	10/2/20	shoreline survey	41° 41' 34.6524"	-71° 21' 46.1016"
Rocky Point, Warwick	RP	6/3/21	sediment grab	41° 41' 27.3006"	-71° 21' 6.357"
Quonset, North Kingstown	Q	6/3/21	sediment grab	41° 34' 18.48"	-71° 23' 46.3194"
Wickford Harbor	WH	10/29/20	sediment grab	41° 33' 51.5694"	-71° 25' 52.7232"
Weaver Cove, Portsmouth	WC	10/16/20	shoreline survey	41° 34' 39.885"	-71° 17' 22.131"
Sandy Pt. Beach, Portsmouth		10/23/20	shoreline survey	41° 33' 8.7906"	-71° 14' 8.055"
Narragansett Town Beach	NB	9/25/20	shoreline survey	41° 26' 8.7468"	-71° 27' 7.92"

Supplementary Table S2. Filter blanks analyzed from air (laminar flow hood), sodium iodide (NaI), and DI water.

Blank ID	Blank Type	Fibers	Fragments	Films	Plastic Sum	Blank notes
1A	Laminar flow hood air	2	0	0	2	black fiber; white fiber
1N	NaI	0	0	0	0	
1D	DI	0	0	0	0	
1	Maximum daily correction	2	0	0	2	black fiber; white fiber
2A	Laminar flow hood air	1	0	0	1	orange fiber
2N	NaI	0	1	0	1	clear fragment
2D	DI	0	0	0	0	
2	Maximum daily correction	1	1	0	2	orange fiber; clear fragment
3A	Laminar flow hood air	3	0	0	3	white fiber; purple fiber; yellow fiber
3N	NaI	0	0	0	0	
3D	DI	0	0	0	0	
3	Maximum daily correction	3	0	0	3	white fiber; purple fiber; yellow fiber
4A	Laminar flow hood air	0	0	0	0	
4N	NaI	0	0	0	0	
4D	DI	1	0	0	1	green fiber
4	Maximum daily correction	1	0	0	1	clear fiber; green fiber
5A	Laminar flow hood air	2	0	0	2	yellow fiber; orange fiber
5N	NaI	1	0	0	1	purple fiber
5D	DI	0	0	0	0	
5	Maximum daily correction	3	0	0	3	yellow fiber; orange fiber; purple fiber
6A	Laminar flow hood air	1	0	0	1	pink fiber
6N	NaI	0	1	0	1	black fragment
6D	DI	0	0	0	0	
6	Maximum daily correction	1	1	0	2	pink fiber; black fragment
7A	Laminar flow hood air	2	0	0	2	red fiber; white fiber
7N	NaI	0	0	0	0	
7D	DI	0	0	0	0	
7	Maximum daily correction	2	0	0	2	red fiber; white fiber
8A	Laminar flow hood air	1	0	0	1	blue fiber
8N	NaI	0	1	0	1	red fragment
8D	DI	0	0	0	0	
8	Maximum daily correction	1	1	0	2	blue fiber; red fragment
	**Total particles**	14	3	0	17	
	**% of total**	82	17.6	0		
	**Maximum daily correction**	3				
	**Average Blank**	2.13				
	**Standard Deviation blank**	0.64				
	**Samples with no correction needed**	42				
	**Samples corrected**	41				
	Notes:					
	Maximum of 2 particles removed during correction for a single sample					

Supplementary Table S3. Pre- and post-blank subtracted microplastic counts.

Sample Information			Sample Counts					Blank Counts						Corrected Counts			
Site	Transect	Sample Type	Fibers	Fragments	Films	Total	Corresponding Blanks ID	Fibers	Fragments	Films	Total	Blank matches	Blank match notes	Fibers	Fragments	Films	Total
Bold Point	1	Grab Sample	121	174	4	299	7	2	0	0	2	1	white fiber	120	174	4	298
	1	Lower Beach	39	64	0	103	7	2	0	0	2	2	red fiber; white fiber	37	64	0	101
	1	Mid-Beach	NA	NA	NA	0	7	2	0	0	2	0	NA	0	0	0	0
	1	Subaqueous Zone	NA	NA	NA	0	7	2	0	0	2	0	NA	0	0	0	0
	1	Upper Beach	191	332	0	523	7	2	0	0	2	2	red fiber; white fiber	189	332	0	521
	3	Lower Beach	38	40	0	78	7	2	0	0	2	1	red fiber	37	40	0	77
	3	Mid-Beach	NA	NA	NA	0	7	2	0	0	2	0	NA	0	0	0	0
	3	Subaqueous Zone	NA	NA	NA	0	7	2	0	0	2	0	NA	0	0	0	0
	3	Upper Beach	773	1357	13	2143	7	2	0	0	2	2	red fiber; white fiber	771	1357	13	2141
Crescent Park	1	Grab Sample	49	55	4	108	8	1	1	0	2	0	NA	49	55	4	108
	1	Lower Beach	18	38	5	61	1	2	0	0	2	2	black fiber, white fiber	16	38	5	59
	1	Mid-Beach	19	36	7	62	1	2	0	0	2	2	black fiber, white fiber	17	36	7	60
	1	Subaqueous Zone	13	43	3	59	1	2	0	0	2	1	white fiber	12	43	3	58
	1	Upper Beach	18	38	5	61	3	3	0	0	3	1	white fiber	17	38	5	60
	2	Lower Beach	19	26	2	47	3	3	0	0	3	0	NA	19	26	2	47
	2	Mid-Beach	23	75	13	111	3	3	0	0	3	0	NA	23	75	13	111
	2	Subaqueous Zone	10	50	4	64	3	3	0	0	3	0	NA	10	50	4	64
	2	Upper Beach	11	46	7	64	3	3	0	0	3	1	white fiber	10	46	7	63
	3	Lower Beach	7	28	9	44	3	3	0	0	3	0	NA	7	28	9	44
	3	Mid-Beach	24	102	37	163	3	3	0	0	3	2	white fiber; yellow fiber	22	102	37	161
	3	Subaqueous Zone	12	55	3	70	3	3	0	0	3	1	white fiber	11	55	3	69
	3	Upper Beach	18	19	1	38	4	1	0	0	2	0	NA	18	19	1	38
Fields Point	1	Lower Beach	21	39	6	66	5	3	0	0	4	0	NA	21	39	6	66
	1	Mid-Beach	34	39	3	76	5	3	0	0	4	1	yellow fiber	33	39	3	75
	1	Subaqueous Zone	69	124	4	197	5	3	0	0	4	2	yellow fiber; orange fiber	67	124	4	195
	1	Upper Beach	43	66	11	120	5	3	0	0	4	0	NA	43	66	11	120
	2	Lower Beach	55	75	6	136	5	3	0	0	4	1	yellow fiber	54	75	6	135
	2	Mid-Beach	23	62	9	94	5	3	0	0	4	0	NA	23	62	9	94
	2	Subaqueous Zone	29	74	1	104	5	3	0	0	4	0	NA	29	74	1	104
	2	Upper Beach	24	56	11	91	5	3	0	0	4	0	NA	24	56	11	91
Grab Samples	1	Grab Sample	69	38	0	107	8	1	1	0	2	1	red fragment	69	37	0	106
	15	Grab Sample	35	19	2	56	8	1	1	0	2	1	blue fiber	34	19	2	55
	2	Grab Sample	21	28	1	50	8	1	1	0	2	0	NA	21	28	1	50
	3	Grab Sample	79	34	0	113	8	1	1	0	2	1	blue fiber	78	34	0	112

Supplementary Table S3. continued:

Sample Information			Sample Counts					Blank Counts						Corrected Counts			
Site	Transect	Sample Type	Fibers	Fragments	Films	Total	Corresponding Blanks ID	Fibers	Fragments	Films	Total	Blank matches	Blank match notes	Fibers	Fragments	Films	Total
Narragansett Town Beach	1	Lower Beach	27	35	8	70	1	2	0	0	2	0	NA	27	35	8	70
	1	Mid-Beach	38	77	19	134	1	2	0	0	2	2	black fiber, white fiber	36	77	19	132
	1	Subaqueous Zone	17	11	0	28	1	2	0	0	2	0	NA	17	11	0	28
	1	Upper Beach	6	10	2	18	1	2	0	0	2	0	NA	6	10	2	18
	2	Lower Beach	46	9	1	56	1	2	0	0	2	2	white fiber, black fiber	44	9	1	54
	2	Mid-Beach	24	59	9	92	1	2	0	0	2	1	black fiber	23	59	9	91
	2	Subaqueous Zone	14	10	0	24	1	2	0	0	2	0	NA	14	10	0	24
	2	Upper Beach	28	16	3	47	1	2	0	0	2	1	black fiber	27	16	3	46
	3	Lower Beach	16	10	0	26	1	2	0	0	2	0	NA	16	10	0	26
	3	Mid-Beach	43	29	6	78	1	2	0	0	2	2	black fiber, white fiber	41	29	6	76
	3	Subaqueous Zone	16	8	0	24	1	2	0	0	2	0	NA	16	8	0	24
	3	Upper Beach	23	6	1	30	1	2	0	0	2	1	black fiber	22	6	1	29
Rocky Point	1	Grab Sample	95	52	3	150	8	1	1	0	2	0	NA	95	52	3	150
	1	Lower Beach	15	22	0	37	5	3	0	0	4	0	NA	15	22	0	37
	1	Mid-Beach	27	49	13	89	5	3	0	0	4	1	yellow fiber	26	49	13	88
	1	Subaqueous Zone	21	48	1	70	5	3	0	0	4	0	NA	21	48	1	70
	1	Upper Beach	19	42	3	64	5	3	0	0	4	0	NA	19	42	3	64
	2	Lower Beach	18	33	1	52	5	3	0	0	4	0	NA	18	33	1	52
	2	Mid-Beach	23	33	1	57	5	3	0	0	4	0	NA	23	33	1	57
	2	Subaqueous Zone	9	20	0	29	6	1	1	0	2	0	NA	9	20	0	29
	2	Upper Beach	22	50	3	75	6	1	1	0	2	1	black fragment	22	49	3	74
	3	Lower Beach	24	67	5	96	6	1	1	0	2	1	pink fiber	23	67	5	95
	3	Mid-Beach	34	60	14	108	6	1	1	0	2	1	black fragment	34	59	14	107
	3	Subaqueous Zone	25	24	0	49	6	1	1	0	2	0	NA	25	24	0	49
	3	Upper Beach	27	53	4	84	6	1	1	0	2	1	black fragment	27	52	4	83
Sandy Point	1	Lower Beach	21	33	3	57	4	1	0	0	2	0	NA	21	33	3	57
	1	Mid-Beach	20	11	4	35	4	1	0	0	2	1	green fiber	19	11	4	34
	1	Subaqueous Zone	13	22	0	35	4	1	0	0	2	0	NA	13	22	0	35
	1	Upper Beach	29	23	1	53	4	1	0	0	2	0	NA	29	23	1	53
	2	Lower Beach	14	26	2	42	4	1	0	0	2	0	NA	14	26	2	42
	2	Mid-Beach	22	14	3	39	4	1	0	0	2	0	NA	22	14	3	39
	2	Subaqueous Zone	17	28	1	46	4	1	0	0	2	0	NA	17	28	1	46
	2	Upper Beach	20	17	0	37	4	1	0	0	2	0	NA	20	17	0	37
	3	Lower Beach	23	21	2	46	4	1	0	0	2	0	NA	23	21	2	46
	3	Mid-Beach	20	31	5	56	4	1	0	0	2	1	green fiber	19	31	5	55
	3	Subaqueous Zone	14	20	0	34	4	1	0	0	2	0	NA	14	20	0	34
	3	Upper Beach	16	28	2	46	4	1	0	0	2	0	NA	16	28	2	46
Weaver Cove	1	Lower Beach	14	17	2	33	2	1	1	0	2	1	clear fragment	14	16	2	32
	1	Mid-Beach	33	37	11	81	2	1	1	0	2	1	clear fragment	33	36	11	80
	1	Subaqueous Zone	23	12	1	36	2	1	1	0	2	0	NA	23	12	1	36
	1	Upper Beach	33	9	4	46	2	1	1	0	2	1	clear fragment	33	8	4	45
	2	Lower Beach	24	12	1	37	2	1	1	0	2	2	clear fragment; orange fiber	23	11	1	35
	2	Mid-Beach	26	30	7	63	2	1	1	0	2	1	clear fragment	26	29	7	62
	2	Subaqueous Zone	25	20	0	45	2	1	1	0	2	1	clear fragment	25	19	0	44
	2	Upper Beach	26	24	2	52	2	1	1	0	2	0	NA	26	24	2	52
	3	Lower Beach	22	15	3	40	2	1	1	0	2	1	clear fragment	22	14	3	39
	3	Mid-Beach	33	21	2	56	2	1	1	0	2	2	clear fragment; orange fiber	32	20	2	54
	3	Subaqueous Zone	20	19	0	39	2	1	1	0	2	1	clear fragment	20	18	0	38
	3	Upper Beach	27	37	5	69	2	1	1	0	2	1	clear fragment	27	36	5	68

a

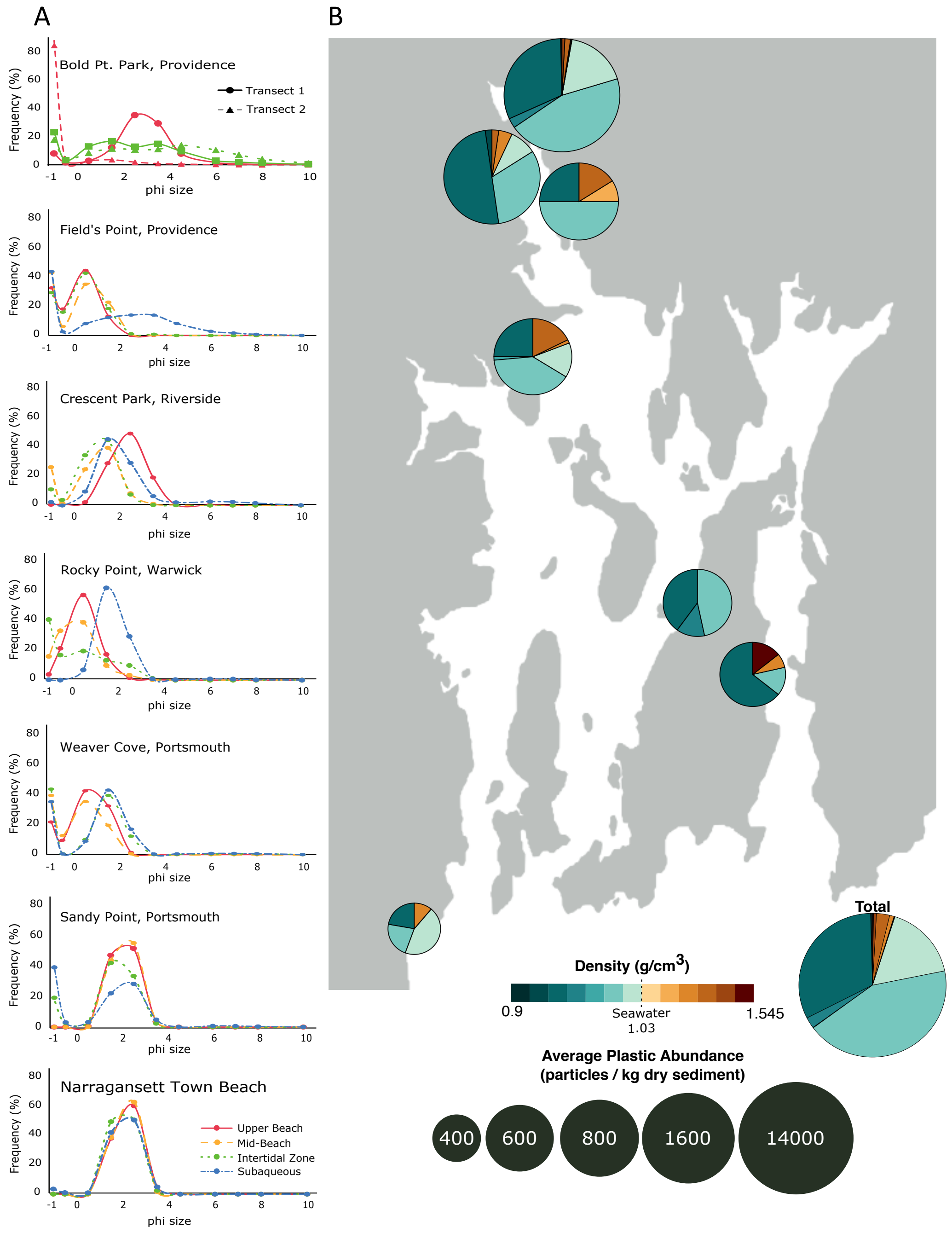

Supplementary Figure S2. Grain size frequency distributions (phi size) for each beach zone (upper, middle, intertidal, subaqueous) were variable between sites (A). Abundance of different plastic types also varied by site (B). The size of the dot represents microplastic concentration at that site (particles / kg dry sediment). The color represents both the particle type and the corresponding particle density. Particles less dense than seawater (<1.03 g cm^{-3}) are shown in green and blue shades. Particles more dense than seawater (>1.03 g cm^{-3}) are shown in shades of brown. The overall frequency for all sites and sampling zones is shown in the "Total" pie chart in the lower righthand corner. Map was created using a grid from Global Multi-Resolution Topography Synthesis (GMRT)[100].

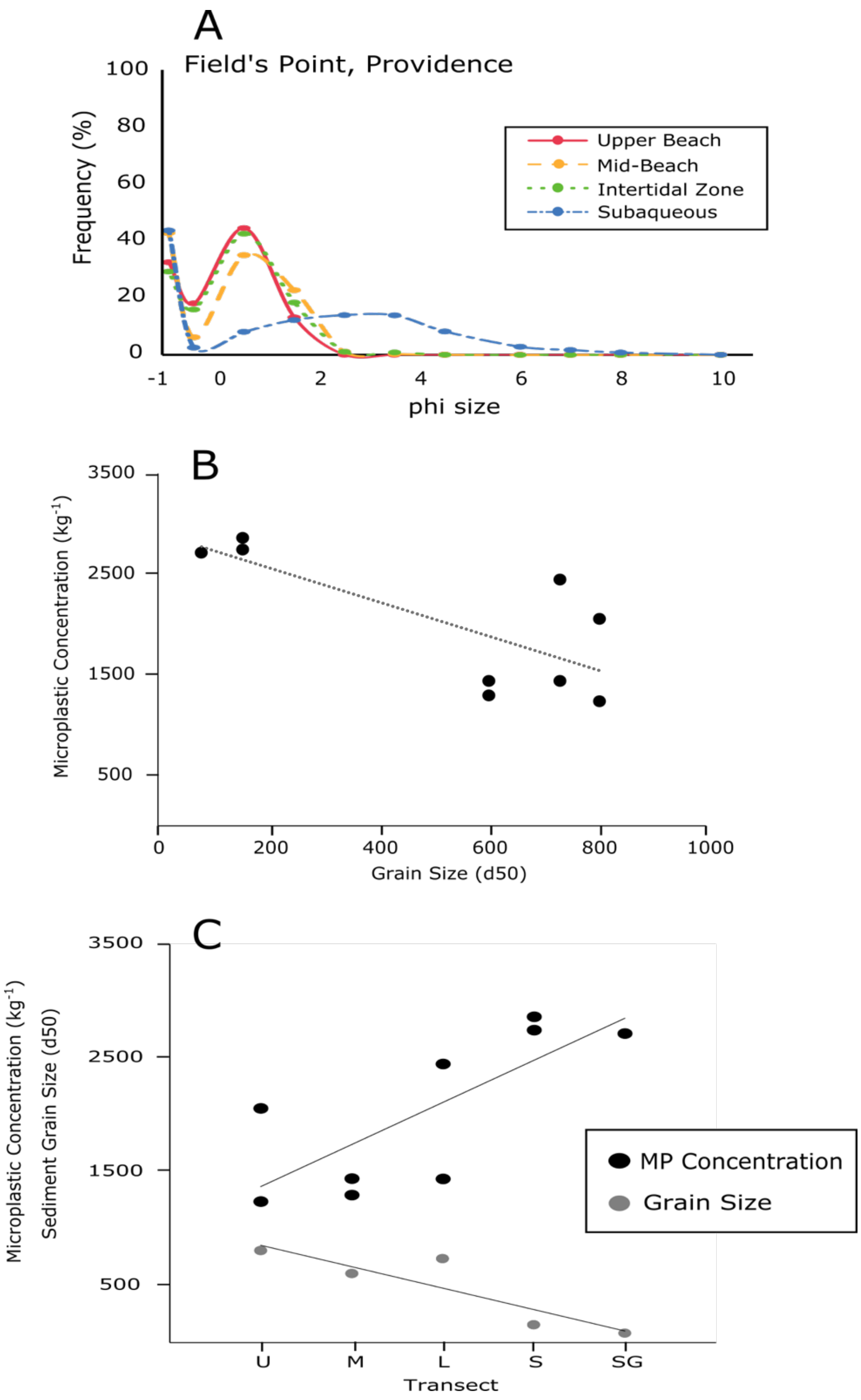

Supplementary Figure S3. Sediment grain size data for Field's Point showed a fining trend from the upper beach to the subaqueous zone (A). Microplastic concentrations decreased as sediment grain size increased (B, $R^2 = 0.56$). Thus, the fining sediment trend (C; gray dots; $R^2 = 75$) coincided with an increased trapping of microplastics (C; black dots; $R^2 = 58$). (U = upper beach; M = mid-beach; L = lower beach; S = subaqueous zone; SG = subaqueous offshore grab sample)
